# Towards An Agency Turn in Animal Law

**DOI:** 10.1093/ojls/gqaf024

**Published:** 2025-07-23

**Authors:** Visa A J Kurki, Paulina Siemieniec

**Keywords:** agency, animal law, legal philosophy, jurisprudence, ethics

## Abstract

The article proposes an agency turn in animal law, following in the footsteps of the political agency turn in animal ethics. The law currently operates on the assumption that animals are passive non-agents, which is reflected in the nature of their legal representation as voiceless and incompetent. We challenge this assumption by identifying three alternative standards for legally representing animals and their interests in the decision-making processes that affect them. According to the: (i) Interest Representation Standard, the best interests of animals are considered; (ii) Listening Standard, animals have a voice, and their input is solicited; and (iii) Empowerment Standard, animals are enabled to make decisions with legal effect. Each standard involves a varying degree of human and animal involvement in legal decision making. It is argued that scholars should reassess the assumption that animals are passive, and seriously consider the extent to which animal law could move towards an agency-affirming paradigm.

## 1. Introduction

In the Western tradition of moral, political and legal philosophy, it is standard to bifurcate parties into active and passive roles, where active parties are seen as decision makers or agents and passive parties are seen as merely recipients of actions, provisions or protections. This division is expressed in, for instance, the distinction between moral *patients* and moral *agents*, between political *wards* and political *agents*, as well as in the legal distinction between *sui iuris* (agents who can act on their own behalf) and *alieni iuris* (beings such as infants, who always require legal representatives). Agency, on the dominant understanding in the moral, political and legal tradition, is thought of as an internal capacity that one either does or does not possess, rather than also as a spectrum of variable ability that is dependent in part on socially and materially enabling factors.

In this active/passive dichotomy, non-human animals (hereafter animals) have widely been seen as passive, lacking the relevant kind of agency in virtue of not being human.^1^ We label this perceived passivity of animals as *the Passivity Assumption*. This assumption has been the point of departure in almost all animal protection legislation and the vast majority of animal law scholarship. Typically, animal protection legislation is premised on *welfarism*, the idea that animals can be exploited and harmed by humans so long as certain welfare standards are upheld and the animals are not subjected to cruelty. Welfarism is premised on the assumption that animals are passive beings, with no legally significant agency of their own to account for. The neglect and suppression of animal agency within this legal doctrine can easily lead to the misrepresentation and substitution of animal interests for our own, thereby heightening their vulnerability to mistreatment. It therefore works in our favour, and against animals, to dismiss them as non-agents.

In this dismissal of animal agency, humans are not only making a *knowledge* claim—that we know what is best for animals—but also a *power* claim about who gets to have a say in the first place.^2^ It is in this sense that Dinesh Wadiwel argues that human sovereignty over animals *appears* to precede ethics. For this reason, ‘ethics constructed after sovereignty works only to regulate or mitigate the violent effects of that sovereignty, while leaving the basic structure of domination intact’.^3^ Legal welfarism can thus be understood as a form of ethical action that is constrained and determined by humans, who are in a position to continue giving themselves legal permission to use animals.^4^ How are animals supposed to make themselves and their claims heard if, as Eva Meijer points out, the formal political (and, we would add, legal) ‘discourse is constructed so that nonhuman animals are deprived of the possibility to address damages done to them on a very fundamental level’?^5^

What this means for the law’s regulation of human–animal interactions is that there are very few and limited exceptions to the fact that even the most progressive legal reform agendas to date do not include animal agency in their purview. For example, the new 2024 animal protection provision in the Belgian Constitution focuses exclusively on ‘the protection and well-being of animals as sentient beings’ and makes no mention of animal agency. Animal passivity is also the standard assumption in more ambitious proposals, such as the Finnish proposal for constitutional animal rights, drafted by the Finnish Animal Rights Law Society.^6^ Our concern with how the law governing animals currently operates is not simply that it is based on the uncritical assumption that animals are passive non-agents, but that even some of the most progressive proposed legal reforms are also starting from the same premise. This has largely gone unnoticed due to the naturalisation and normalisation of the Passivity Assumption about animals. One potential risk of this is that legal systems and institutions are bound to remain anthropocentric if animals continue to be marginalised and feature passively, rather than as agents who might be enabled by humans in certain respects to play some part in reshaping the laws that they are governed by.

In recent years, political philosophers have increasingly acknowledged animal agency. This emerging discourse is the direct result of the political turn in animal ethics,^7^ where the Passivity Assumption is critically interrogated, and the question of animal agency is being revisited. Most political philosophers, especially those outside animal studies, adopt the Passivity Assumption about animals as well as human children and people with severe cognitive disabilities. However, there are some notable exceptions, like Sue Donaldson and Will Kymlicka, who promote a more expansive conception of agency that feminist and disability thinkers have developed. This conception encompasses a wider range of political actors and activity. Donaldson and Kymlicka argue for an inclusive conception of citizenship that extends the right to active political participation to domesticated animals because of their membership in society and claims as stakeholders.^8^

Others, such as Robert Garner, Angie Pepper and Richard Healey—while sceptical of some of the more far-reaching claims of the aforementioned authors about the *political* agency of animals—have put forward ideas that nevertheless challenge the Passivity Assumption insofar as they affirm the *non-political* agency of animals.^9^ The ongoing debate about how to define political agency and whether animals possess it indicates that the issue of animal agency is being taken seriously.

The animal agency debate has been relatively limited in legal scholarship, though legal scholars such as Charlotte Blattner, Michaël Lessard, Janneke Vink and Katharina Braun have explored the topic from different perspectives.^10^ We will explore some of their ideas over the course of this article.

In this article, we identify three standards, two of which move beyond the Passivity Assumption about animals in law, towards the possibility of recognising legal animal agency. The Interest Representation Standard recognises that animals have interests and are therefore entitled to having their interests considered, represented and legally protected by humans. The Listening Standard acknowledges that animals have a voice and that their input should be solicited on matters that affect them. Lastly, the Empowerment Standard views animals as agents who can be directly empowered to make or co-make decisions with legal effect.

The goal of this article is not to make an unqualified claim about animal agency, but rather to critically interrogate the Passivity Assumption from which the theory and practice of animal law operate. If, as Meijer suggests, it is indeed the case that ‘animals are individuals with their own perspectives’,^11^ then it would seem best to approach them as such and take their contributions seriously.

Philosophers arguing for animal agency in politics are not claiming that animals are capable of exercising political agency independently, that is, without the necessary accommodations or supports in place, especially not in the ‘formal’ realms of politics, which are made by and for humans.^12^ They acknowledge that support and accommodation are required. With this in mind, animal agency should not be disregarded before we consider the role of support and accommodation in the facilitation of agency more broadly and the role that it could play for animals. In other words, we would be mistaken to equate the unmet need for support and agency with incapacity.^13^ Indeed, the opportunity for animals to meaningfully exercise agency is frequently denied before the possibility is even investigated.

Assuming that some party—a child, a person with disabilities or an animal—is passive goes hand in hand with the ‘need’ for wardship models whereby a guardian makes decisions in the name of their ward. However, as Donaldson and Kymlicka flag, such models ‘systematically overestimate the willingness and capacity of guardians to act in the interests of the vulnerable, and systematically underestimate wards’ capacities and contributions’ in addition to ‘the distorting effects of standpoint, self-interest, and hierarchy’.^14^ These risks have already been acknowledged in disability law, which has increasingly moved away from the Interest Representation Standard, but this has yet to be explored in the case of animal law. While we do not seek to provide a definitive answer about whether animals are legal agents, we do offer a framework for thinking about animal legal agency.

The article is organised as follows: section 2 examines the pervasive Passivity Assumption about animals in law, highlighting how the traditional understanding of legal agency has expanded to include human groups that were once excluded but not animals—at least, not yet. Section 3 concerns the political agency debate about animals, highlighting how the concept of political agency has been defined, critiques of capacity-based perspectives and the key implications for law. Section 4 develops a framework for recognising animal agency in law by proposing three standards for understanding the nature of animal legal representation and participation.

## 2. Agency in Law

Legal as well as political and moral philosophy all operate on some understanding of agents and non-agents. It is useful to, first, distinguish between the *requirements* and *stakes* of some type of agency. An *agency requirement* is a criterion that some entity must meet in order to be classified as an agent in some context. A *stake* is what follows from being classified as an agent. For instance, in moral philosophy, the requirements for moral agency are often set quite high, presupposing, for instance, the capacity to deliberate upon moral reasons. Whether such a high bar is problematic depends (at least partly) on the stakes of moral agency: if only moral agents are entitled to moral consideration, then we will end up excluding various groups, such as animals, from moral consideration. However, if we accept that there are also moral patients, then the stakes of moral agency are not about moral considerability but rather about moral responsibility.

In law, there is no general established discourse that would distinguish between agents and non-agents.^15^ Rather, there are various discourses and taxonomies that implicitly trade on the agent/non-agent distinction, such as doctrines determining who is criminally liable, who can decide to enter contracts, who can consent to medical procedures, and so on.

We do not aim to offer a general theory of legal agency, but will rather focus on animals. For the purpose of understanding legal animal agency in particular, we can discern two main categories: (i) legal regulation of animals’ bodily liberty; and (ii) giving legal significance to animals’ actions (and omissions). Both involve some recognition of animals’ agency, though such recognition is not always positive for the animal.

First, the law structures and constrains animal agency by norms pertaining to human control over animals’ bodily liberty. For instance, law may permit the extreme limitation of the liberty of sows in gestation crates, whereas the bodily liberty of some protected wild animals, such as lynxes, might be protected relatively robustly, eg under the EU Habitats Directive.^16^ Both cases contain an implicit recognition that animals are agents: the sows are kept in gestation crates in order to *control* their agency and *minimise* their bodily liberty, whereas the regulation of lynxes' agency is much more focused on *ensuring* their bodily liberty (within certain parameters).^17^

Second, the law can give legal significance to animals’ actions. In this case, the law will take some act or omission of an animal as an ‘input’ that will feed into legal considerations. Again, such recognition of animal agency might not be in place for animal-friendly reasons.

One example where an exercise of animal agency may function as ‘input’ are situations where animal actions may trigger legal consequences, ie changes in some parties’ rights and duties. For instance, a dog’s attacking a human being may trigger the latter’s permission to defend themself, and tort liability for the owner of the dog.^18^ The capacity to trigger such consequences by volitional acts may be labelled ‘wide powers’.^19^ The distinctive feature of wide powers is that the obtaining legal consequences need not be related to the agent’s desires or intentions in any way: the dog need not attack with the intention of triggering tort liability in order to bring about said consequence. We are again dealing with a doctrine that, while recognising animal agency, is not in place for animal-friendly reasons.

However, a particularly interesting subgroup of wide powers is the *legal recognition of one’s will and preferences*. It is this category in particular that we will be focusing on, and when we speak of legal agency in the rest of the article, we mean solely this aspect of agency. In the human case, the traditional gold standard is the legal decision-making power that is typically called ‘legal power’, ‘legal capacity’ or ‘legal competence’. Entering a contract would be a classic example of the exercise of this capacity. However, we will discuss below other ways in which law can give recognition to one’s will and preferences.

We will now look more carefully at how law’s agency requirements have shifted in the recent decades: the will and preferences of previously excluded groups of human individuals, such as children and people with disabilities, have gained increasing prominence.

Western law has traditionally distinguished between those seen as capable of making decisions and ‘the incapable’.^20^ This categorisation has been based on a demanding and individualistic understanding of agency, which has often denied the agency of people with cognitive disabilities and children^21^—as well as animals. We can, roughly, distinguish two rationales for the partial or full denial of legal agency to the individuals in these groups.

The first justification is paternalistic:^22^ if children or people with cognitive disabilities could freely make legally valid decisions, they might easily make *bad decisions*. Hence, they need to be protected from themselves, as well as from those who might seek to take advantage of them.

The second rationale is conceptual. Whereas, say, 12-year-old children are protected from making bad decisions, it is quite likely that infants *cannot* make legally enforceable decisions—good or bad. According to standard jurisprudential accounts of legal competence/power, one must be able to perform acts with *legal intentions*, ie the intention to produce legal effects, in order to be able to hold competences. Hence, entering a contract requires the capacity to intend to enter a contract. Given that infants presumably lack such an understanding of the normative reality, they cannot be given competences to decide about their own affairs.

In spite of these paternalistic and conceptual considerations, a number of solutions have been developed to increase the legal agency of children and people with cognitive disabilities. These developments have been advanced by two important international conventions: the Convention on the Rights of the Child and the Convention on the Rights of Persons with Disabilities.^23^

Even though the primary consideration under the Convention on the Rights of the Child is still the ‘best interests’ of the child, Article 12 also gives children the right to be heard, and their views shall be given ‘due weight in accordance with [their] age and maturity’.^24^ Children here are not necessarily treated as decision makers in their own right, but they are involved in the processes pertaining to them in a new way and ascribed a voice. On one hand, they can provide information about the matter; on the other, their views also have a weight in themselves. Children have also increasingly been given direct legal decision-making power. For instance, in many jurisdictions, a child can refuse a vaccination if deemed sufficiently mature to understand its significance.^25^ Hence, children are increasingly included in the Listening and Empowerment Standards (which will be elaborated on in section 3).

The Convention on the Rights of Persons with Disabilities, on the other hand, is primarily about the empowerment of people with disabilities. Whereas the Convention on the Rights of the Child requires a more nuanced assessment of an individual child’s capacities, the approach of the Disability Convention is based on a more relational understanding of disability and decision making: a person with disabilities, who would traditionally have been considered incompetent to make decisions, is instead provided support mechanisms which enable them to make decisions. It is this relational approach to agency, prevalent in disability studies, that has recently been picked up in the political agency debate over animals. We will address this debate in the following section.

Now, while traditional boundaries of legal agency have been challenged both in the case of children and in people with disabilities, the Passivity Assumption still prevails in animal law scholarship: the overwhelming—and almost unquestioned—consensus has been that animals must be treated as passive subjects of law, represented by human beings, rather than as agents capable of making decisions about their situation. We can take the conceptual debate over whether animals can hold rights as an example. Theories of rights have mostly been divided into two groups: will theories and interest theories.^26^ According to the will theory, holding a right presupposes the capacity to actively exercise it, whereas for the interest theory, being a passive beneficiary is sufficient for counting as a potential right holder. As Serrin Rutledge-Prior notes, rights theorists ‘have long been united’ in believing that ‘while the interest theory can accommodate the rights of nonhuman animals, the same is not true of the will theory’.^27^ This is why legal scholarship on animal rights has broadly resorted to interest-based accounts of rights in order to argue for animal rights. In 1993, Gary Francione took the appointment of guardians, representing an animal’s best interests, to be the ‘standard response’ to the problem that ‘only humans can resort to the use of courts to enforce their rights’.^28^ Thirty years later, it is still the standard response to this problem, and the standard approach within animal law more generally.^29^

Though animal law scholarship and theory has not yet extensively or explicitly engaged with the question of animal agency, the issue has received much more attention in political theory and philosophy. It is to this discussion that we now turn.

## 3. On the Political Agency Debate about Animals

Over the last decade or so, animal ethics has taken a political turn. The political agency debate is one example of this, as it concerns the inclusion of animals and their interests in democratic theory and practice. Political philosophers in this debate agree that animals require representation but disagree on the nature of their representation, that is, whether they would occupy the political status of agency or should be relegated to passive wardship.

The disagreement stems from how the concept of political agency is understood. Defining political agency in cognitivist terms, as a type of human capacity that animals do not possess, implies that animals would only qualify for rights to consideration because they are deemed politically capable.^30^ If, however, political agency is not merely a capacity internal to individuals, but also a relational process and distributed phenomenon, then we can begin to explore the possibility of humans and animals engaging in joint politics. Put another way, this would open the door for doing politics with animals, as opposed to them being passively represented by us.^31^

Section 2A outlines the relevant terms employed by political animal philosophers. Section 2B will cover some critiques of the capacity-based definition of political agency. Section 2C will note some key takeaways for initiating a similar agency turn in animal law.

### A. Conceptualising Political Agency

Although the Passivity Assumption about animals remains the prevailing position in political philosophy, generally speaking, it has recently been challenged from two different angles: (i) the capacity-based view of political agency; and (ii) the relational and distributed understanding of agency. This subsection briefly examines these two definitions of agency and their application to animals.

Theorists in the first camp extend the traditional definition of political agency as a highly specific and demanding type of linguistic and cognitive human capacity. This basic idea is captured by Pepper, who argues that political agency requires the following ‘individually necessary, and jointly sufficient capacities: (i) the capacity to intend to affect institutions; (ii) the capacity to engage in complex forms of shared intentionality; and (iii) the capacity to imagine alternative political futures’.^32^ On the capacity-based view, animals do not qualify as political agents and therefore cannot possess the ‘special rights, powers, and responsibilities’ that are associated with political agency ‘such as the rights to political participation and freedom of speech’.^33^ Theorists in this vein generally concede that animals are agents, though not political agents. Animals, in this view, would have a democratic right to ‘political consideration’ but not political participation because they are not seen as capable of representing themselves.^34^ These political animal philosophers challenge the idea that animals are passive by recognising their agency, even if that agency is not regarded as politically significant.

The possibility of doing politics with animals has only been investigated by a small number of theorists. In their extensive body of work on animal citizenship, Donaldson and Kymlicka have consistently argued for the legal and political recognition of domesticated animals as our co-citizens on the basis of their social membership.^35^ Given their prominence in this regard, we will focus primarily on their work as an exemplary account of political animal agency. Donaldson and Kymlicka argue that

the underlying moral purpose of citizenship … is to ensure that all members of the community have a chance to act on their own understanding of their subjective good, to exercise meaningful forms of control in their lives, and to participate in shaping the society and laws by which they are governed.^36^

For them, the right to enact substantive citizenship should track social membership. Every member of society, including domesticated animals, should have the right to participate politically through some mode of self-representation.^37^ Donaldson and Kymlicka contend that the capacity for ‘independent political agency via rational speech’ should not be a prerequisite for inclusion in democratic participation and to argue otherwise is to misunderstand the very nature and purposes of citizenship.^38^

In their earlier work, Donaldson and Kymlicka^39^ turned to feminist and disability articulations of dependent agency^40^ and supported decision making^41^ to explore the possibility of relating to domesticated animals as active political participants. Donaldson and Kymlicka extended these models of agency and mechanisms for participation, which are steadily supplanting paternalistic frameworks of guardianship and rule, to include domesticated animals. Concretely, this means that humans are responsible for domesticated animals in both private and public spheres as beings with individual and collective scripts of ‘the good’.^42^

In their more recent work, Donaldson and Kymlicka^43^ draw on Sharon Krause’s relational and distributed account of agency to argue that animals have the capacity to act and participate politically. For Krause, agency is not reducible to intentional choice and control over action; rather, it consists of two components: *subjectivity or selfhood* and *efficacy*.^44^ Agency minimally requires subjectivity because being an actor is not about merely being a cause of outcomes, but about embodying and expressing ‘self-directed modes of action’.^45^ As Krause explains, agency is a foundational concept that tells us what it means to attribute politically and morally significant activity to the subject from whom it originates.^46^

Anchoring agency in selfhood or subjectivity is therefore essential to conceptually distinguishing between agential and non-agential activity/actors. Hence, non-sentient things and natural forces do not meet the subjectivity requirement and cannot be held morally or otherwise accountable for actions because ‘their’ actions are not truly ‘theirs’. The *efficacy component* concerns the social and material distribution of agency, which is a dimension of agency that the capacity-based theorists largely dismiss.^47^ It is in this way that Krause understands agency to be ‘the affirmation of one’s subjective existence through concrete action in the world’ because its actualisation partly depends on the right conditions.^48^

Donaldson and Kymlicka reject the Passivity Assumption by arguing that animals can be enabled as political agents ‘when their social and material environments provide uptake of their subjectivity and serve as the holders of their identities’.^49^ ‘Uptake’ here refers to an affirming response by others to the individual initiation of action and a holding environment that materially supports agency.^50^ Meijer also rejects said assumption, arguing that animals are not politically incapable; humans rarely interpret the things that animals do and communicate as political or politically relevant.^51^ This raises the question of how much human interference obstructs the realisation of animal agency.

A second problem raised by Donaldson and Kymlicka against the capacity model of political agency is that it divides members of society along the lines of activity and passivity—a ruling class of political actors that makes decisions for themselves *and* on behalf of political patients.^52^ Stacey Clifford Simplican calls this the ‘capacity contract’, which is a type of domination contract.^53^ Donaldson and Kymlicka conclude that the capacity-based approach to politics is antithetical to democracy because it promotes the rule of one group over another.^54^ In fact, Donaldson and Kymlicka claim that the capacity contract has even been applied to cognitively and linguistically able human groups to exclude them and render them politically incapable.

Third, Donaldson and Kymlicka argue that wardship fails to adequately safeguard against the bias and self-serving nature of power holders’ knowledge regarding what they claim is in the ward’s best interests.^55^ These concerns are echoed by Meijer: ‘The epistemic problem with humans representing non-human animal interests on the basis of expert knowledge is that this knowledge is partly shaped by centuries of oppression and unequal power relations.’^56^ The solution for these thinkers lies in exploring the possibility of political animal agency, as it offers a pathway to equal and effective self-governance—a possibility that the capacity-based view precludes.^57^

Finally, Paulina Siemieniec has argued that the construal of political agency as a paradigmatically human capacity can be *dehumanising* for those who are seen as deficient in it because of how closely possessing the capacity for political agency is tied to being seen as human.^58^ This view of not ‘normally able’ individuals as less-than-human is dually subhumanising for animals because they are not human or capable in a ‘normally able human’ sense.^59^

The point of considering the critiques of the capacity-based view of political agency is not to convince the reader to adopt one concept of political agency over another. Indeed, we return to Pepper’s work on (non-political) animal agency later, in section 3, to argue that the ideas of consent, assent and dissent can be useful for thinking about legal animal agency. It is significant that both camps of political philosophers agree that animals are agents, even if they disagree about the type of agency animals are capable of. For the purposes of this article, the conceptual distinction between the competing definitions of agency is not as useful as extracting the methodology for approaching the issue of animal agency, and what this could mean for starting a legal animal agency debate.

### B. Political Agency Debate: Takeaways for Law

In this subsection, we will consider some areas of potential overlap for the political agency debate’s methodological import to the legal context as well as a dissimilarity in terms of conceptual content. As political animal philosophers acknowledge, humans will inevitably play a crucial role in the representation of animals and their interests, even on the more ambitious accounts of agency where theorists make the ‘radical’ argument that animals should have rights to indirect representation by human trustees *and* political self-representation.^60^

For instance, Meijer discusses the role of humans in translating and/or interpreting the political voice and agency of animals as well as human–animal interactions institutionally.^61^ The author suggests that this can be done with the aim of progressively developing new forms of representation and new types of human–animal legal and political institutions and interactions.^62^ Donaldson and Kymlicka also concede that human representatives will have to play a role in enabling the political participation of domesticated animals. But this ‘role needs to be embedded within a larger story of animals’ direct political voice and participation’.^63^ Rather than assuming animals are voiceless, this position starts from the opposite assumption. This is significant because animals are ‘destined to be silent in political matters’, so long as they are considered mute.^64^ The same goes for animal voice and participation in law.

Political animal philosophers in the other camp, who do not believe that animals can politically represent themselves, promote a trustee model of wardship that is passive and indirect. It is worth noting, however, that this position is also clearly better than the norm of completely discounting animals and their interests. As Donaldson and Kymlicka comment, ‘institutional reforms that require representing and counting animals’ interests in political decision-making could [in and of themselves] be truly revolutionary’.^65^ In this way, both camps of political animal philosophers have pushed the discourse beyond the traditional Passivity Assumption about animals. The key difference between these two camps is that, unlike the proponents of the capacity-based view, those who espouse the distributed and relational account of agency believe that politics is not something that is merely done to or on behalf of animals, but something that animals can also do and participate in.^66^

What is the relevance of this for the discussion of legal animal agency? The political agency debate about animals has at least three takeaways for animal law. First, animal law scholars should examine their assumptions about the preconditions of agency. Are certain capacities being construed as prerequisites to the exercise of legal agency, or are enabling social and material conditions being acknowledged as playing a significant role in shaping agency and determining its effectiveness? In other words, scepticism over animals’ capacity to exercise legal agency should not simply rest on the unexamined *assumption* that animals are passive non-agents.

Legal scholars and practitioners should recognise that the effective exercise of agency hinges on uptake. It would be mistaken to equate this need for enabling conditions with the incapacity to exercise agency. Indeed, the groups that are most frequently denied agency are the same ones that are not provided with adequate support and accommodation for the uptake of their agency.^67^ The presence or absence, as well as the extent, of agency-enabling mechanisms and resources should be factored into assessments of agency. This, of course, would require an expanded understanding of agency that encompasses such considerations.

Second, there should be an openness to exploring, and potentially re-engineering, relevant jurisprudential concepts and categories to better accommodate animal agency and the various ways that it is embodied and expressed by different kinds of animals individually and collectively. If traditional accounts of, say, consent cannot accommodate animal consent, these accounts should be re-examined. The political agency debate can be instructive here, but its upshot for law should be considered carefully. For example, the capacity-based view is often associated with a denial of animals’ *political* agency, but there might in fact be some room for their *legal* agency even on the capacity-based view if we are open to a more careful assessment of animals’ capacities. Consenting to a medical procedure is an example of how the threshold for legal agency may be lower than for political agency on a capacity-based view: consenting likely does not require the capacity to imagine alternative political futures, which Pepper posits as necessary for political agency.^68^ However, such consenting may still be considered an instance of legal agency. Therefore, some of the criteria for political agency may be irrelevant or inapplicable for certain instances of legal agency.^69^

Third, potential instances of animal legal agency could be found in places that legal scholars do not traditionally associate with agency. Animal voice and participation in law would require actually listening and paying attention to what animals are saying through their embodied modes of communication and rethinking how and where decisions are made so that animals can be meaningfully included in these processes.^70^ Animals usually make decisions in what are deemed as informal contexts^71^ that may nevertheless have legal significance. An example of this is in wildlife conservation, where migratory animals’ decisions, such as altered migration patterns in response to environmental changes, can prompt the creation of new protected areas or adjustments to wildlife management laws.

This brings us to our next section, where we identify three standards of legal representation for animals. Each standard highlights an important dimension of legally representing animals and their interests, including their interest in agency when it is applicable and appropriate to do so. *For both humans and animals*, legal agency may involve different kinds of representation, depending on the context and decision at hand. As we will see, the three standards are informed by certain methodological aspects of the political turn in animal ethics, such as resisting the Passivity Assumption and shifting standards. We tread lightly into the new terrain of legal animal agency by suggesting three standards for thinking about how animals and their interests might be represented in decision making.

The nature of one’s legal representation is partly determined by the specific type of decision being made. There are some instances in which self-representation is not applicable for humans or animals, irrespective of and aside from individual capacity. The three paradigms that we outline in the next section are meant to illuminate how we can begin to shift away from the Passivity Assumption, even in situations where it is truly necessary and appropriate to make decisions on behalf of others.

## 4. Legal Agency for Animals: Three Standards

How might the developing ideas of animal agency be applied in the legal realm, then? As was noted above, there have already been legal developments where legal agency has been extended to new categories of human beings: children and people with disabilities. We will now consider the potential for such developments in the context of animal law. The discussion will be centred around three standards: the Interest Representation Standard; the Listening Standard; and the Empowerment Standard.

From the outset, it is important to mention that the three standards are highly contextual: an individual may be a part of each of them in different legal contexts. Consider, for instance, a 15-year-old human child in an imaginary legal system. His legal situation can very well be a mix of the three standards:

- his parents can pick a school for him, based on his best interests (Interest Representation Standard);
- if his parents get divorced and have a custody dispute, the court will ask him for his opinion on which parent he wants to live with, but the court’s ultimate decision will also be based on other factors (Listening Standard); and
- he can only be given vaccinations with his consent (Empowerment Standard).

It is worth thinking about how the legal situation of an animal might also be a mix of the three standards. How legal decisions are categorised matters because, without these categories, it is easy to ignore the possibility of listening to or empowering animals in legal decisions. For instance, if we assume that all legal decisions involving animals fall under the Interest Representation Standard, then they will never get a chance to be represented otherwise. Take, for instance, the case of a dog in an animal shelter, in an imaginary legal system:

- the shelter staff may pick which family adopts him, in accordance with his best interests (Interest Representation Standard);
- if a couple taking care of him get divorced and have a custody dispute, the court will ‘ask’ him for his opinion on which human he wants to live with, but the court’s ultimate decision will also be based on other factors (Listening Standard); and
- he may not be forced to work against his will (Empowerment Standard).

We will explore the framing of these ideas in more depth below, using concrete examples to illustrate the distinctive features of each legal representation standard.

### A. Interest Representation Standard

For most of Western legal history, animals have been viewed simply as commodities, lacking any legal protection. As was briefly noted above, even under this regime, which treats animals like commodities, law has in fact implicitly recognised animals’ agency in certain legal doctrines, such as when regulating the harm caused by animals’ actions. However, the purpose of such regulation of animal agency has not been to protect animals’ interests or agency. Most legal systems have moved away from treating animals as mere commodities and introduced at least some legal protections for animals. As noted above, almost all of the existing animal protection legislation is founded upon the dominant welfarist paradigm of animal law, focusing on the protection of animals from cruelty, as in the affliction of ‘unnecessary’ suffering.^18^

Under welfarism, some animal interests are legally protected, but these protections tend to be defined in terms of what is in the ‘best interests’ of animals relative to human interests. Animals are denied agency in decision making on the assumption that they are incapable of having a say or directly participating in such processes. Consequently, human representation of animals and decision making on their behalf are viewed as pragmatically necessary.

Since animals are not regarded as having individual preferences or agency that are legally relevant, humans resort to what we believe is in their best interests. Thus, representation of ‘interests’ here refers to what is objectively good for a subject, or what is perceived as such by the subject’s representatives. In other words, there is no involvement of the subject in the interpretation of their interests or contestation thereof.^72^

Nearly all existing animal protection legislation has relied on this paternalistic idea of protecting animal interests. We thus classify welfarist approaches primarily under the Interest Representation Standard, though not necessarily so. The same standard has mostly been taken as the default starting point for more ambitious proposals, drafted by animal lawyers, seeking to safeguard animals more comprehensively. For instance, the proposal for a Convention on Animal Health and Protection (under the auspices of the Global Animal Law Association) is based on the principle that ‘the interests and needs of non-human animals are to be fully considered in every field of human endeavour’.^73^ Similarly, the Finnish proposal for constitutional animal rights is not welfarist but, rather, based on a fundamental-rights-based framework. Regardless—with some exceptions, which will be discussed below—the proposal is primarily situated within the Interest Representation Standard, making, for instance, reference to the principle that ‘The interests and individual needs of animals must be taken into account in all private and public activities that have a significant impact on their living conditions or chances of survival’.^74^

The Interest Representation Standard is the standard approach, and it can serve as a framework for animal rights law. However, it should not be seen as the only, *unquestionable* option. Similar to how the consideration rights camp of political animal philosophy operates, according to Meijer:

Proponents of animal rights want to establish just treatment for other animals by instituting laws that would protect their basic liberties. In this scheme, humans would design the laws involved, and other animals would depend on humans to interpret these laws and speak for them. Even proposals that ask for extensive reformulations of animal representation—for example, in the form of trustees or proxies such as official advocates or ombudspersons supplemented by institutionalized systemic accountability by ethological experts, media, animal advocates and others who speak for animals—still start from the idea that humans should act on behalf of non-human animals, and do not involve any justification of their acts to the animals they represent.^75^

One obvious problem with the Interest Representation Standard is that it assumes that humans always know what is ‘objectively’ in the best interests of animals. However, as Meijer argues, individual humans and animals possess the most intimate and accurate knowledge about themselves and are therefore ‘in the best epistemic position’ to determine their subjective good.^76^ That which is not captured by the ‘objective’ evaluation of animal interests is precisely the sort of information that may influence the direction of a decision.

We are sympathetic to the concerns that Meijer and others raise regarding animal agency and epistemic justice, which is why we discuss two legal animal agency-affirming standards below. But this does not imply that the Interest Representation Standard should be completely discarded in the legal context of animal agency. In both the human and animal cases, there are situations where the Interest Representation Standard is required, appropriate, and permissible. For instance, many legal systems recognise that there are certain circumstances in which individuals cannot consent to a life-saving procedure, but healthcare providers can perform them anyway based on the principles of necessity or best interests. This is especially clear in emergency situations where immediate medical intervention is required to prevent death or serious injury.

Similarly, individual animal choices could contradict what is in their best interests due to a communication barrier. It would be difficult, if not impossible, for humans to explain to animals that a procedure is lifesaving or medically necessary because humans do not usually understand or talk in the languages that animals use. Due to this communication barrier, an animal’s physical resistance to a life-saving procedure does not mean that the treatment is not in their best interest or that the animal prefers to die. The resistance may stem from the conditions surrounding the procedure—such as the animal feeling uncomfortable or scared in a new environment with unfamiliar sounds, smells and strangers. Animals may be resisting because they do not understand why they are being physically restrained or that the procedure will improve or save their life.

As we will see, the key to determining which of the three standards of legal representation is appropriate lies in the contextual analysis and categorisation of legal animal agency and decision making. The next two standards focus on decision-making processes where human evaluations of what is best for an individual or group of animals can be influenced or supplemented by animal agency. In other words, these decisions are not based solely on what is perceived as being in the ‘best interest’ of animals, but also include the input and involvement of the animals themselves.

### B. Listening Standard

Under the Listening Standard, animals still have human representatives, but it is the task of the representatives to ascertain animals’ preferences, needs and interests by seeking to communicate with them (typically in a non-verbal, embodied manner).^77^ This standard differs from the Interest Representation Standard in two key respects: procedurally and substantively.

First, the *method* of making decisions is different: we seek to make decisions based on interactions and in consultation with the party in question. We may, for instance, learn that the preferences and interests of an animal may differ from the interests that are typical of their species. A static notion of ‘species norms’^78^ could prevent us from noticing or trying to solicit the preferences of individual animals, which may be considered atypical for their species.^79^

The second difference pertains to *what* we base the decision on: at least some weight should be put on the personal preferences of animals, rather than what we might take to be in their objective interest.^80^ For example, we might argue that it is in the ‘best interests’ of unhoused dogs to be housed, but it could also be in the idiosyncratic interests of some unhoused dogs to remain free-roaming.

According to the Listening Standard, humans reserve the right to make the ultimate decisions, and the animal’s input and preferences are only one consideration. This feature of the Listening Standard is clearly visible in the realm of children’s rights: the Convention on the Rights of the Child requires that children be heard before various decisions, but the children’s views are simply seen as one consideration. This is what distinguishes the Listening Standard from the Empowerment Standard, which makes the decisions of the subject determinative of some outcome (by, for instance, prohibiting medical procedures performed against the subject’s will).

Any move towards a recognition of animal agency will encounter the question of whether animals are capable of ‘understanding all of the material information relevant to any particular choice of action’, as Healey and Pepper put it.^81^ However, as they note, such capabilities are best ‘assessed relative to specific domains, decisions, or interactions’.^82^ The Listening Standard is easiest to imagine in small-scale contexts. There are, in fact, some such individual cases where companion animals have (putatively) been heard. For instance, in the Spanish case of *Indie*, the eponymous dog was ‘heard’, meaning that the dog’s behaviour was observed by the judge in order to determine for which of the parties she felt more affection.^83^ It is certainly plausible that a dog could understand what life with either ex-spouse A or ex-spouse B would entail and could therefore form a preference regarding which one to live with. Hence, the dog’s capabilities would meet Healey and Pepper’s requirements. Of course, there are difficult epistemological problems in how such preferences should be ascertained.

For a larger-scale example of interacting with animals as collectives, we turn to Meijer’s discussion of a 2014 conflict between seagulls and humans in the Netherlands, which they describe as a material form of deliberation that could inform legislation.^84^ The case involves seagulls displaced by habitat destruction, which led them to relocate to urban areas, where they became disruptive to humans. A Dutch politician responded by proposing to lower legal protections and shoot the birds. This was opposed by a bird protection organisation, which instead suggested non-lethal alternatives, such as experimenting with stronger plastic bags, providing the birds with natural food sources and creating designated islands for them to nest. The seagulls’ responses were observed, and the measures were modified accordingly. Meijer views this human–animal interaction as an example of the kind of interspecies collective decision making that can be explored through experimentation.

The seagull example is primarily about political rather than legal agency. However, it highlights a key dimension of the Listening Standard, which shifts the epistemological source of decision making to animals as agents in shared *dialogical (back-and-forth)* processes of decision making—even if the ultimate decision still lies with human beings. A defining feature of the Listening Standard is that it involves a multidirectional or cyclical, as opposed to a unidirectional or linear, movement of power by listening and being responsive to what animals are telling and showing us through their actions.

The Listening Standard can be seen as either radical or disappointingly conservative, depending on our perspective. It is radical in that it does indeed recognise animals as agents whose expressed and embodied preferences are relevant. However, it can be seen as not going far enough if we do not view the solicitation of input at the listening stage as actually handing over the decision-making power to animals. This leads us to ask if decision-making power can be shared with animals in the first place and, if so, how.

### C. Empowerment Standard

This subsection considers whether and how animals could also be included in the Empowerment Standard. Here, animals would be viewed and treated as actual decision makers, capable of making legal decisions on the basis of their wills and preferences.

In the realm of disability law, human beings with cognitive disabilities have—as a result of the Convention on the Rights of Persons with Disabilities—experienced a shift from substituted to supported and shared models of decision making, or what we refer to as a transition from the Interest Representation Standard to the Empowerment Standard. The shift has led to the creation of legal frameworks that recognise individuals’ agency while providing the necessary mechanisms for support, assistance and representation. However, animals are not yet on the radar of such expansions.

The distinction between the Listening and Empowerment Standards can be elucidated using the distinction that Daniel Groll makes between *substantive relevance* and *structural decisiveness*. If one’s views or preferences are substantively relevant, they will be ‘one factor among others that goes into an all-things-considered judgment’.^85^ They will weigh in favour of some outcome. Let us take a situation where we are considering whether to perform some medical procedure on a dog named Dizzy, even though she is resisting the procedure. Under the Listening Standard, Dizzy’s resistance to the procedure could be a reason for not performing the procedure. However, this reason would not necessarily be a *sufficient* reason, and it could be outweighed by whatever reasons there are in favour of performing the procedure, such as Dizzy’s improved health. Under the Empowerment Standard, on the other hand, Dizzy’s opposition to a specific type of work, for instance, would be an ‘authoritative demand’: it would ‘trump whatever other considerations are on the table’.^86^ Hence, Dizzy would essentially possess the power to veto any involuntary employment. A similar example can be given in the case of non-domesticated animals vetoing captivity by resisting, escaping or evading capture. Now, whether animals can, conceptually, issue authoritative demands is a difficult philosophical question.

The Empowerment Standard does not automatically preclude the use of representatives. Even paradigmatic legal agents, such as adult human beings ‘of sound mind’, often use representatives (for instance, when appearing in court). However, they can normally choose their representatives themselves.^87^ At any rate, we will first consider the issue of animals’ making legal decisions directly, without representatives.

Having legal competences (ie legal powers), meaning the capacity to make decisions with legal effect, has traditionally been seen as central for legal agency. Such competences certainly lie at the heart of the Empowerment Standard, at least in its classical form. However, there has been plenty of scepticism about whether competences could be extended to animals. A standard understanding of competence requires what may be called *legal intentions*: the intention to change one’s and others’ legal rights and duties.^88^ For instance, legal contracts are normally valid only if the parties intend to be legally bound by them, and consenting to a medical procedure means waiving one’s right to bodily integrity.^89^ It is doubtful whether animals can understand ideas such as LAW or LEGAL RIGHT. This is why Hillel Steiner—defending a version of the will theory of rights—classifies animals as ‘unempowerable’. He writes:

[T]he set of creatures who are capable of exercising powers [ie competences] can never amount to more than a small subset of the creatures who have interests, however interests are construed. Hence foetuses, minors, the comatose … *to say nothing of the members of virtually all other known species*—must all lack Will Theory rights.^90^

Some scholars working in animal ethics have made similar arguments. For instance, in their discussion of animal consent, Angie Pepper and Richard Healey argue that:

While it is ultimately an empirical question, it seems unlikely that many animals will have (i) the concepts of RIGHT, CONSENT, or PERMISSION (ii) the knowledge of the relevant normative facts (eg that they have certain rights against us, that these rights can be waived through certain intentional acts of consent), and (iii) the ability to form and communicate the relevant complex intentions concerning such concepts.^91^

However, this traditional conceptualisation of the limits of empowerable parties has received an increasing amount of resistance, visible in both legal and scholarly developments. The criticism has been particularly pronounced in the field of disability law. As noted above, the Convention on the Rights of Persons with Disabilities is based on a new model of supported decision making, which empowers people with cognitive disabilities to make decisions about their lives by extending them a sufficient amount of support. Importantly, this shifts the focus from the individual and their capacities to the interaction between individuals and the relational and material conditions that affect their agency, and can be adjusted to be more accommodating and enabling.^92^

Regardless, many are sceptical that animals could be endowed with legal competences, such as the competence to consent.^93^ For instance, Lauren van Patter and Charlotte Blattner have discussed how the requirement of voluntary participation might be operationalised in the case of research using animal participants. They write:

Voluntary participation means that animals cannot be forced to participate in research. Our framework for voluntary participation combines ongoing embodied assent from animal participants and mediated informed consent from a human representative. This combined model is based on the premise that although many animals are able to understand, weigh, and communicate decisions in the context of research, if the broader context, purpose, and implications of research remain inaccessible to them, we cannot consider them to be informed consenters to the extent typically required.^94^

However, drawing on the work of Tumilty and others, they argue that animals should always provide their embodied assent, ‘established through a non-human animal’s willingness to undertake tasks associated with the research’.^95^ The suggested model would thus comprise a mix of the Interest Representation Standard and a version of the Empowerment Standard. Similar ideas, based on the concepts of assent and dissent, have been proposed by Healey and Pepper, who suggest that the requirement of assent would give the animal normative authority.^96^ Katharina Braun labels such conceptual proposals as ‘concept-related concepts’.^97^ This approach has in fact been employed in one part of the Finnish constitutional proposal, which is otherwise primarily based on the Interest Representation Standard: section 4 of the proposal prohibits the insemination of an animal ‘against the animal’s will’.^98^

Successfully assenting or dissenting does not presuppose an understanding of law or norms. However, one worry from a more general legal-theoretical perspective is how to place this in the broader theory of legal powers and competences. For instance, Rutledge-Prior discusses broader ways of understanding these categories. She suggests an interpretation of the will theory of rights presupposing

that the rights-holder has preferences about how they are treated and is able to clearly express these preferences in an effort to affect a change (or maintain consistency) in their situation—even if they don’t comprehend how these preferences might take shape in the legal sphere.^99^

This account seems to have some connection to Kurki’s suggestion that the law could recognise *non-legal* but regardless *normative* intentions.^100^ Rutledge-Prior notes that ‘There is mounting evidence that many animals can and do develop expectations about how they are treated’.^101^ For instance, one famous experiment done by Frans de Waal and Sarah Brosnan involved the experimenters’ offering monkeys either cucumber or grape in exchange for tokens. If a monkey was given a piece of cucumber but saw another receive a grape, she would likely reject the cucumber.^102^ The experiment is normally presented as evidence of monkeys’ sense of inequality. However, it can also be seen as suggesting that the monkeys could understand the idea of a transaction, and perhaps even money. Even if they might not be able to understand the *abstract* idea of a legal system, they do seem to be able to function using more concrete concepts that are highly analogous to those employed within law. The legal system could then treat such transactions as legally enforceable, at least to some extent (by, for instance, protecting the contractual and property rights of monkeys *vis-à-vis* humans). Kurki has labelled such a potential category of competences *soft competences*.^103^ However, relying solely on soft competences in expanding empowerment to animals would likely be highly limited in scope, extending only to animals whose normative agency we can recognise and therefore enforce.

In conclusion, the traditional views of legal authority—often connected to the will theory of rights—have seen animal empowerment as a conceptual impossibility, but some potential solutions have been developed to meet these challenges, even if there is likely still some conceptual work to be done.

### D. The Three Standards: A Summary

As summarised in Table 1, what makes the Empowerment Standard distinct from the Listening Standard is the level of authority granted to an animal’s will and preferences. Under the Listening Standard, an animal’s preferences are considered but can be outweighed by other considerations, while under the Empowerment Standard, the animal’s preferences are treated as authoritative, giving them the power to veto decisions and actively shape or influence decisions that affect them. The information gathered from the animal in question therefore plays a different role: when we *listen* to an animal’s voice, their input is solicited so that they can be part of the deliberations involved in a legal decision-making process; when animals are *empowered* as legal actors, the exercise of their will is respected, protected and enabled—at least on a small scale. However, offering general guidelines for choosing between the three standards is difficult.

**Table 1.** The standards of animal representation in law

Standard	Characterisation	Examples where potentially appropriate
Interest	Representation of animal interests by humans	Where being heard and empowered is inappropriate or inapplicable (eg due to communication barriers, lacking capability or more fundamental interests at stake, such as life or liberty)
Listening	Solicit input from animals, who have a right to be considered	Family law cases (divorce, adoption and ‘guardianship’, custody and access disputes); health and medical decisions about animals
Empowerment	Enable animals to make legal decisions	To veto involuntary work and captivity

However, both the Listening and Empowerment standards carry risks. As Rutledge-Prior puts it, we should be aware of ‘the exploitative power structures that shape many human–animal interactions and that might foreclose the possibility of meaningful consent (and other ethical exchanges) within the context of these interactions’.^104^ For example, if a horse needs to be ‘broken’ to be ridden, this seems to suggest that the horse’s will to not be ridden by a human is disrespected. Whether it is through harsh or gentler means, the horse is commanded to submit to and obey the human rider, who essentially takes over and controls the horse’s body and movements (pace, direction, etc). This is a clear case where the will of the horse is gradually worn down through forced submission. The process can lead to what is termed ‘adaptive preferences’: the horse might now ‘prefer’ to be ridden, even if this preference is the result of extensive manipulation and coercion. If, after this process, we then give the horse the authority to refuse being ridden, this ‘veto right’ is part of the Empowerment Standard, but only in a nominal sense. This is why the Empowerment Standard is predicated on a meaningful range of opportunities and options for the exercise of choice to be relatively free. Similarly, Rutledge-Prior suggests that ‘animals held in factory farms and invasive biomedical research centers simply cannot consent to their treatment, given their cognitive, emotional and social capacities have been so severely hampered’.^105^ Hence, extending the Empowerment Standard to such contexts could be essentially empty. At least, it should be accompanied by interest-or listening-based safeguards, as in van Patten and Blattner’s proposal above, where a human representative would need to provide informed consent. Braun, on the other hand, proposes that ‘guardians making decisions (while considering animal assent and dissent as a matter of animal wellbeing) should be the rule, while decisions based on animals’ expression of subjective preferences should be the exception’.^106^ This is essentially a combination of the Listening and Empowerment standards: the main rule requires listening to animals by taking their assent and dissent into account, whereas empowerment is the occasional exception.^107^

In situations where the Empowerment Standard is not appropriate, the most agency-affirming choice may be one that maximises their agency in future scenarios. This approach may be especially relevant for animals coming out of abusive or exploitative conditions that have largely denied them agency. In these cases, a decision that aims to maximise their future agency in areas like choice, movement and social association is likely to provide the best possible outcome—one they would plausibly choose for themselves. It is in this way that the standards come back full circle, as there are situations in which what is in the best interests of an animal may be the most agency-affirming option available.

## 5. Conclusion

The idea that animals have interests and that those interests require legal protection is now widely accepted among animal law scholars and practitioners. However, the current paradigm of animal protection assumes that animals are not legal actors but passive beings, incapable of representing themselves, either as individuals or collectives. This is true in at least two senses: (i) in the assumption that animal interests can, or should, only ever be represented by humans; and (ii) in the consistent denial of their agency as having little to no legal bearing in decision-making processes.

Anthropocentric legal systems are not presently designed, equipped or meant to protect some of the most basic interests of animals, let alone recognise their agency. Efforts to strengthen the legal protection of animals nevertheless persist, and *how* animals are represented matters.

Animal welfare laws protect animal interests to the extent that they do not contradict the dominant social, cultural and economic norms surrounding the human use of animals. When coupled with the Passivity Assumption about animals, legal welfarism can easily allow humans to make false claims about the animals on whose behalf they speak and act, thereby misrepresenting them and their interests—portraying them as non-existent or non-essential—to serve an extractive agenda. We argued that these claims are not just epistemic, asserting that humans know better than animals, but also about the largely uncontested power to override animal interests and agency.

As a result of the Passivity Assumption, animal law remains firmly rooted in the Interest Representation Standard, where animal agency has yet to be acknowledged. In other words, animal law scholars and practitioners are focused on the legal protection and representation of animal interests without even considering the possibility that there might be certain legal contexts in which animals could play an active role in shaping decisions.

As we have shown, this does not imply that the Interest Representation Standard would not play a role in an agency-affirming paradigm. In both the human and animal cases, there are situations where the Interest Representation Standard is the most appropriate option. For instance, in many legal systems, performing a life-saving procedure on an individual who cannot consent to it would be considered appropriate and permissible because it is necessary and in their best interests, even if this person is regarded as a legal agent.

But, as we have argued, it would be misguided to treat *all* legal cases involving animals as cases where humans ought to represent animals as passive beings. For this reason, we proposed two additional standards—the Listening Standard and Empowerment Standard—to explore legal animal agency and reimagine the nature of legal representation as a role within which animals can participate more directly and actively. Our argument is that, as a rule, when animals’ input is relevant to legal decisions, their voices should be solicited and factored into those decisions. Moreover, in cases where animals can be enabled to make legal decisions—individually or collectively—humans should create the necessary social and material conditions to facilitate legal animal agency by removing barriers and providing appropriate support and accommodations.

The next step is to ask what our political and legal institutions and relationships would look like if they were redesigned to affirm, rather than deny, animal agency. Properly recognising legal animal agency requires dismantling and rebuilding certain social and material aspects of our existing legal and political systems. Partly, this involves questioning whose agency is currently supported and how these disparities reinforce oppressive hierarchies, and then addressing them by implementing legal and political frameworks that empower the animals whose agency they presently disable.

Finally, a focus on animal agency could also offer a new locus for animal rights arguments. As Michaël Lessard notes, animal sentience—the dominant premise of animal rights scholarship—has gradually become recognised in legal documents and doctrines, but this recognition has not yielded tangible changes in the legal status of animals. Legally recognising animal agency could be an alternative pathway for improvement of the legal status of animals, partly because respect for agency has relatively intuitive normative implications.^108^

Consider the case of the 43 young female rhesus macaques that escaped from Alpha Genesis Inc, a research and medical testing laboratory in Yemassee, South Carolina, where they had been subjected to invasive experiments. This incident, which occurred in November 2024, sparked a public debate about whether these monkeys now had a legal claim to freedom, ie to remain free after escaping their captors. This outcry is an example of the intuitive attractiveness of a focus on agency: when given the chance, the rhesus macaques escaped captivity and freed themselves, which many members of the public could appreciate.

However, the story also functions as a cautionary tale. Since this was a case involving captive wild animals, their captor no longer has ownership over the escaped monkeys, especially if they join a community of wild monkeys. The monkeys joined a free-roaming community of rhesus macaques that has been residing on what is known as ‘Monkey Island’, the state’s Morgan Island, for over four decades. In recent years, Alpha Genesis Inc, one of largest monkey breeding operations in the world, has landed a federal contract to manage the ‘free-range’ monkey colony. While Alpha Genesis Inc is responsible for feeding the monkeys and providing them with veterinary and other care services, the facility is at liberty to pluck monkeys from the island for medical and research testing. All 43 rhesus macaques that escaped in November 2024 were actually taken from the island and recaptured: they were lured back to captivity with peanut butter and jelly sandwiches and biscuits, after two months of roaming freely in the woods. Now, did they thereby *assent* to more testing? Claiming as much would clearly be spurious. Hence, the case highlights the need for a careful design of legal animal agency frameworks; simply relying on, say, animal assent would be vulnerable to exploitation.

Recognising animal agency challenges foundational assumptions in current animal law frameworks, highlighting the urgent need for a paradigm shift. The example of the rhesus macaques vividly demonstrates both animals’ active participation in shaping their lives and the legal inadequacies in acknowledging their agency. Moving forward, embracing new standards could transform animal law from mere representation to genuine inclusion. Such an evolution would acknowledge animals not merely as passive objects of human action, but as actors, communicators and decision makers.

## Footnotes

1 We talk of ‘animals’ here mostly as a single category, while of course acknowledging the immense variety among animals.

2 Paulina Siemieniec, ‘A Political Theory of Accessibility and Care for Animals’ (PhD thesis, Queen’s University at Kingston 2025).

3 Dinesh Wadiwel, *The War Against Animals* (Brill 2015) 22.

4 ibid 22.

5 Eva Meijer, ‘Interspecies Democracies’ in Bernice Bovenkerk and Jozef Keulartz (eds), *Animal Ethics in the Age of Humans: Blurring Boundaries in Human–Animal Relationships* (Springer 2016) 58.

6 Finnish Animal Rights Law Society, ‘Proposal for the Constitution of Finland: Chapter XX On the Fundamental Fights of Animals’ <www.elaintenvuoro.fi/english/> accessed 15 January 2025. Visa Kurki, one of the authors of this article, is also one of the drafters of the proposal.

7 See Sue Donaldson and Will Kymlicka, *Zoopolis* (OUP 2011); Alasdair Cochrane, *Animal Rights Without Liberation* (Columbia UP 2012); Robert Garner, *A Theory of Justice for Animals* (OUP 2013); Tony Milligan, ‘The Political Turn in Animal Rights’ [2015] Politics and Animals 6; Lucy Parry, ‘Deliberative Democracy and Animals: Not So Strange Bedfellows’ in Robert Garner and Siobhan O’Sullivan (eds), *The Political Turn in Animal Ethics* (Rowman & Littlefield 2016); Bernd Ladwig, ‘Against Wild Animal Sovereignty: An Interest‐based Critique of Zoopolis’ (2015) 23 Journal of Political Philosophy 282.

8 Donaldson and Kymlicka, *Zoopolis* (n 7).

9 Robert Garner, ‘Animals and Democratic Theory: Beyond an Anthropocentric Account’ (2017) 16 Contemporary Political Theory 459; Richard Healey and Angie Pepper, ‘Interspecies Justice: Agency, Self-Determination. and Asset’ (2020) 178 Philosophical Studies 1223.

10 Charlotte E Blattner, ‘Turning to Animal Agency in the Anthropocene’ in Bernice Bovenkerk and Jozef Keulartz (eds), *Animals in Our Midst: The Challenges of Co-existing with Animals in the Anthropocene* (Springer International Publishing 2021); Michaël Lessard, ‘Beyond Sentience: Legally Recognizing Animals’ Sociability and Agency’ (2023) 14(1) Journal of Animal Ethics 89; Janneke Vink, *The Open Society and Its Animals* (Palgrave Macmillan 2020); Katharina Braun, ‘Animal Consent and the Ambit of Law’ [2025] Res Publica.

11 Eva Meijer, *When Animals Speak: Toward an Interspecies Democracy* (New York UP 2019) 2.

12 Siemieniec (n 2) 67.

13 ibid 55.

14 Sue Donaldson and Will Kymlicka, ‘Inclusive Citizenship Beyond the Capacity Contract’ in Ayelet Shachar and others (eds), *Oxford Handbook of Citizenship* (OUP 2017) 847, 855.

15 The most established legal use of the terms ‘agent’ and ‘agency’ has to do with representation. For instance, the term ‘law of agency’ normally refers to an area of law regulating the roles of a *principal* and an *agent* (ie the principal’s representative).

16 Council Directive (EEC) 92/43/ on the conservation of natural habitats and of wild fauna and flora [1992] OJ L206/7.

17 This can be seen as an example of what Michaël Lessard calls a ‘narrow interpretation of agency’, which ‘would at least result in a negative obligation not to interfere in the implementation of an animal’s choices’. Lessard (n 10) 98.

18 Visa AJ Kurki, *A Theory of Legal Personhood* (OUP 2019) 74–5.

19 Visa AJ Kurki, ‘Expanding Agency and Borders of Competence’ in Gonzalo Villa-Rosas and Torben Spaak (eds), *Legal Power and Legal Competence* (Springer Cham 2023) 47.

20 Hendrik Verhagen and Laura Macgregor, ‘Agency and Representation’ in Jan M Smits, Jaakko Husa, Catherine Valcke, and Madalena Narciso (eds), *Elgar Encyclopedia of Comparative Law* (Edward Elgar Publishing 2023).

21 At least some children; infants are a clear case.

22 The term ‘paternalistic’ is used here in a neutral, rather than negative, sense.

23 Convention on the Rights of the Child (adopted 20 November 1989, entered into force 2 September 1990) UNGA Res 44/25; Convention on the Rights of Persons with Disabilities (adopted 13 December 2006, entered into force 3 May 2008) UNGA Res 61/106.

24 UNGA Res 44/25.

25 This capacity to consent for minors is occasionally called ‘Gillick competency’ because of the UK case *Gillick v West Norfolk and Wisbech* AHA [1985] UKHL 7.

26 Matthew H Kramer, ‘Rights in Legal and Political Philosophy’ in Gregory A Caldeira, R Daniel Kelemen and Keith E Whittington (eds), *The Oxford Handbook of Law and Politics* (OUP 2008); Matthew H Kramer, ‘Rights Without Trimmings’ in Matthew H Kramer, Nigel E Simmonds and Hillel Steiner, *A Debate Over Rights: Philosophical Enquiries* (OUP 1998).

27 Serrin Rutledge-Prior, ‘Addressing the Will Theory Challenge to Animal Rights’ (2025) 73(2) Political Studies 505, 505. However, “soft” will theories, which allow for representatives, might be able to accommodate the rights of animals.

28 Gary L Francione, ‘Personhood, Property, and Legal Competence’ in Paola Cavalieri and Peter Singer (eds), *The Great Ape Project* (St Martin’s Griffin 1993) 248, 250.

29 For one recent example, see Joshua Jowitt, ‘Legal Rights for Animals: Aspiration or Logical Necessity?’ (2020) 11(2) JHRE 173.

30 Angie Pepper, ‘Political Agency in Humans and Other Animals’ (2021) 20 Contemporary Political Theory 296.

31 Sue Donaldson and Will Kymlicka, ‘Doing Politics with Animals’ (2023) 90(4) Social Research: An International Quarterly 621.

32 Pepper (n 30) 298.

33 ibid 296.

34 Garner, ‘Animals and Democratic Theory’ (n 9); Dan Hooley, ‘Political Agency, Citizenship, and Nonhuman Animals’ (2018) 24 (4) Res Publica 509.

35 Donaldson and Kymlicka, *Zoopolis* (n 7); Sue Donaldson and Will Kymlicka, ‘Unruly Beasts: Animal Citizens and the Threat of Tyranny’ (2014) 47 Canadian Journal of Political Science 23; Sue Donaldson and Will Kymlicka, ‘Rethinking Membership and Participation in an Inclusive Democracy: Cognitive Disability, Children and Animals’ in Barbara Arneil and Nancy J Hirschmann (eds), *Disability and Political Theory* (CUP 2016); Donaldson and Kymlicka, ‘Doing Politics with Animals’ (n 31).

36 Donaldson and Kymlicka, ‘Inclusive Citizenship’ (n 14) 855.

37 Sue Donaldson, ‘Animal Agora: Animal Citizens and the Democratic Challenge’ (2020) 46 Social Theory and Practice 709.

38 Donaldson and Kymlicka, ‘Inclusive Citizenship’ (n 14) 840, 855; Donaldson and Kymlicka, ‘Doing Politics with Animals’ (n 31).

39 Donaldson and Kymlicka, *Zoopolis* (n 7).

40 Anita Silvers and Leslie Pickering Francis, ‘Justice through Trust: Disability and the “Outlier Problem” in Social Contract Theory’ (2015) 116 Ethics 47.

41 Michael J Prince, *Absent Citizens: Disability Politics and Policy in Canada* (University of Toronto Press 2009).

42 Donaldson (n 37).

43 Donaldson and Kymlicka, ‘Doing Politics with Animals’ (n 31).

44 Sharon R Krause, ‘Agency’ (2017) 3 Political Concepts: A Critical Lexicon <www.politicalconcepts.org/agency-sharon-krause/> accessed 17 January 2025.

45 Maureen Sander-Staudt, ‘Reassembling Political Assemblies: Care Ethics and Political Agency’ (2008) 39 Journal of Social Philosophy 269, 270.

46 Krause, ‘Agency’ (n 44) 1.

47 Sharon S Krause, ‘Bodies in Action: Corporeal Agency and Democratic Politics’ (2011) 39 Political Theory 299, 301.

48 Sharon S Krause, ‘Beyond Non-domination: Agency, Inequality and the Meaning of Freedom’ (2013) 39 Philosophy and Social Criticism 187, 197.

49 Donaldson and Kymlicka, ‘Doing Politics with Animals’ (n 31) 640.

50 Donaldson and Kymlicka (building on Krause’s work) define the role of uptake and the material environment as follows: ‘Humans become agents if and when other subjects are willing and able to be the “bearers” and “holders” of our subjectivity, providing “uptake” of our expressions and actions, interpreting, responding, supporting, and extending them. (Consider, for example, how the speech of a high-status person is “taken up” by others in deliberation.) Agency also depends on whether and how the material environment bears our agency. (Consider how an able-bodied person has easier access to the council chamber located up a flight of steps compared to a person using a wheelchair or how the destruction of an open and accessible gathering space for large crowds undermines the possibility of engaging in certain kinds of protest.) In short, agency depends on the “holding environment” and how the social and material context holds people’s identities and subjectivities. Donaldson and Kymlicka, ‘Doing Politics with Animals’ (n 31) 639.

51 Meijer, *When Animals Speak* (n 11).

52 Donaldson and Kymlicka, ‘Inclusive Citizenship’ (n 14) 841.

53 Stacey Clifford Simplican, *The Capacity Contract: Intellectual Disability and the Question of Citizenship* (University of Minnesota Press 2015).

54 Donaldson, ‘Animal Agora’ (n 37) 717.

55 ibid 851.

56 Meijer, *When Animals Speak* (n 11) 204.

57 Donaldson and Kymlicka, ‘Inclusive Citizenship’ (n 14) 853.

58 Siemieniec (n 2) 63. See also Raffael N Fasel, *More Equal Than Others: Humans and the Rights of Other Animals* (OUP 2024) 105.

59 Siemieniec (n 2) 63.

60 Donaldson, ‘Animal Agora’ (n 37) 709.

61 Eva Meijer, ‘Political Communication with Animals’ (2013) 5 Humanimalia 1, 4.

62 ibid 5.

63 Donaldson, ‘Animal Agora’ (n 37) 718.

64 Meijer, ‘Political Communication with Animals’ (n 61) 2.

65 Donaldson and Kymlicka, ‘Doing Politics with Animals’ (n 31) 624.

66 ibid 624.

67 Siemieniec (n 2) 42.

68 Pepper (n 30) 298.

69 Janneke Vink seems to dismiss the possibility of political animal agency, but then draws a similar conclusion (that animals are passive) in the legal sphere as well. This is clear in the parts of her book that align more closely with legal philosophy, especially in her focus on the prospect of constitutional protections and rights for animals. Janneke Vink, *The Open Society and Its Animals* (Palgrave Macmillan 2020) 23–30, 205–335.

70 Sue Donaldson and Will Kymlicka, *‘Political Agency’ in Animals and the Right to Politics* (OUP, forthcoming) ch 5.

71 ibid ch 5.

72 The subject is, at least, not involved in the concrete legal decision. However, animal protection law is partly based on animal welfare science, which may use methods building on animal agency. For instance, so-called preference tests involve having animals choose between different options. Their choices are then used to draw conclusions about their preferences and interests. IJH Duncan, ‘Science-Based Assessment of Animal Welfare: Farm Animals’ (2005) 24(2) Revue scientifique et technique 483.

73 Global Animal Law Association, ‘UN Convention on Animal Health and Protection (UNCAHP)’ 1st pre-draft (2018) 2.

74 Finnish Animal Rights Law Society (n 6) s 1.

75 Meijer, *When Animals Speak* (n 11) 203.

76 ibid 204.

77 The phrase ‘Listening Standard’ is inspired by Mark Rowlands’ phrase ‘listening paradigm’. Mark Rowlands, *Can Animals Be Persons?* (OUP 2019) 199.

78 Martha Nussbaum, *Frontiers of Justice: Disability, Nationality, Species Membership* (Belknap Press 2006).

79 Donaldson and Kymlicka, *Zoopolis* (n 7) 100; if we solicit information from individual animals but only use this information to better understand their individual interests (without giving weight to their preferences), we have a more ‘individualized’ Interest Representation Standard.

80 That is, on the presumably ‘objective’ interests of animals that humans determine or decide, rather than their embodied and expressive subjective interests.

81 Healey and Pepper (n 9) 178. They use the term ‘competence’, but we have opted to use ‘capability’ to avoid confusion with the more strictly jurisprudential idea of competence.

82 ibid 1229.

83 Juzgado de Primera Instancia No 4 [2019].

84 Meijer, *When Animals Speak* (n 11) 212.

85 Daniel Groll, ‘Paternalism, Respect, and the Will’ (2012) 122 Ethics 692, 703.

86 ibid 701.

87 This is not to suggest that those who are viewed (from an ableist perspective) as deviating from the image of the ‘paradigmatic’ human cannot also be involved in choosing their representatives. On the other hand, it is not unheard of that courts appoint legal counsel to an individual against that individual’s wishes. This would be an example of the Interest Representation Standard.

88 See eg Lars Lindahl and David Reidhav, ‘Legal Power: The Basic Definition’ (2017) 30 Ratio Juris 158. The well-established definition of ‘legal power’ by Joseph Raz makes explicit reference not to intentions, but to reasons. However, it does place similar constraints on who or what can hold legal powers. Neil Maccormick and Joseph Raz, ‘Voluntary Obligations and Normative Powers’ (1972) 46 Proceedings of the Aristotelian Society 59.

89 The most important exception to this requirement in common law is labelled ‘reliance’.

90 Hillel Steiner, ‘Working Rights’ in Kramer, Simmonds and Steiner (n 26) 189, 259. However, Steiner emphasises that we regardless ‘have significant moral duties … to protect the interests of these *unempowerable* creatures’ (ibid 259).

91 Healey and Pepper (n 9) 1231.

92 Siemieniec (n 2) 48.

93 See eg Braun (n 10).

94 Lauren E van Patter and Charlotte Blattner, ‘Advancing Ethical Principles for Non-Invasive, Respectful Research with Nonhuman Animal Participants’ (2020) 28 Society & Animals 171.

95 Emma Tumilty and others, ‘Ethics Unleashed: Developing Responsive Ethical Practice and Review for the Inclusion of Non-Human Animal Participants in Qualitative Research’ in Ron Iphofen and Martin Tolich (eds), *The SAGE Handbook of Qualitative Research Ethics* (SAGE Publications 2018) 403.

96 Healey and Pepper (n 9) 1226.

97 Braun (n 10).

98 Finnish Animal Rights Law Society (n 6) s 4.

99 Rutledge-Prior (n 27) 9.

100 Kurki, ‘Expanding Agency’ (n 19).

101 ibid 10.

102 Sarah F Brosnan and Frans BM de Waal, ‘Monkeys Reject Unequal Pay’ (2003) 425 Nature 297, 298.

103 Kurki, ‘Expanding Agency’ (n 19).

104 Rutledge-Prior (n 27) 516.

105 ibid 516.

106 Braun (n 10).

107 Braun does not seem to make the distinction between the Interest Representation and Listening Standards.

108 Lessard (n 10) 91.

